# How to promote exclusive breastfeeding in Ireland: a qualitative study on views of Chinese immigrant mothers

**DOI:** 10.1186/s13006-021-00358-4

**Published:** 2021-01-15

**Authors:** Haoyue Chen, Cheng Li, Qianling Zhou, Tanya M. Cassidy, Katherine M. Younger, Siao Shen, John M. Kearney

**Affiliations:** 1 Department of Maternal and Child Health, School of Public Health, Peking University, Beijing, China; 2 Beijing Nutrition Resources Institute, Beijing Academy of Sciences, Beijing, China; 3 School of Nursing, Psychotherapy and Community Health, Dublin City University, Dublin 9, Ireland; 4 School of Biological Sciences, Technological University Dublin, Kevin Street, Dublin 8, Ireland; 5 The Stony Brook School, Stony Brook, NY USA

**Keywords:** Suggestions, Exclusive breastfeeding, Chinese, Immigrant, Ireland, In-depth interview, Qualitative

## Abstract

**Background:**

The exclusive breastfeeding rate in Ireland is very low with extremely slow annual growth. The population of immigrants in Ireland is increasing. Improving exclusive breastfeeding practice among immigrants may contribute to the overall improvement of exclusive breastfeeding rates in Ireland. This study was conducted to elicit suggestions on improving exclusive breastfeeding rate for the first 6 months among Chinese immigrants in Ireland.

**Methods:**

Fourteen semi-structured in-depth interviews were conducted with Chinese immigrant mothers residing in Ireland, who breastfed exclusively for 4 to 6 months. Interviews were recorded and transcribed in Chinese. Data were analyzed using a qualitative thematic analysis. Themes were developed through categorization of codes and via in-depth discussion between two researchers.

**Results:**

Themes generated from the thematic content analysis were: 1) suggestions for new mothers: being strong mentally and getting support from family and friends; 2) suggestions for employers: creating a supportive workplace by setting up private rooms and breastmilk storage facilities; 3) suggestions for healthcare professionals: advocating breastfeeding in the hospital and addressing cultural differences by recruiting multilingual staff; 4) suggestions for the government: promoting breastfeeding by initiating societal and policy changes.

**Conclusions:**

The key findings emerging from this study may be considered in the development of breastfeeding promotion strategies in Ireland. Our findings could also have implications for other English-speaking countries with low rates of exclusive breastfeeding.

## Background

Appropriate feeding practices are vital for children’s optimal growth and development [1]. Exclusive breastfeeding is promoted worldwide as the ideal way for infant feeding due to its protective effects on infant mortality, overweight/obesity, type 2 diabetes and other chronic diseases [2–7]. The World Health Organization (WHO) suggests exclusive breastfeeding within the first 6 months of life and continued breastfeeding until the age of two or above [8].

It has been reported immigration to another country may have a great impact on breastfeeding initiation and maintenance rates [9–13]. Previous research have shown that immigrant mothers have higher breastfeeding initiation rates and longer duration than non-immigrant mothers, especially those who had a shorter duration of stay in the new country than their country of origin [14]. This phenomenon has been observed in many countries and is widely referred as “the healthy immigrant effect” [9–13]. However, a number of studies reported “the healthy immigrant effect” in breastfeeding might be influenced by the length of residency in the host country [11]. As the length of residency increased, the breastfeeding rates lowers. This might be explained by factors such as acculturation, exposure to the new social norms, barriers to access health services due to language or cultural differences [10, 15, 16].

Additionally, there is some evidence that breastfeeding rates might vary by country of origin. Rio et al. found that Chinese immigrants had a lower prevalence of breastfeeding initiation rates when compared to immigrants from Latin America, Eastern Europe, Maghreb or sub-Saharan African [9]. Goel et al. and Tuttle et al. also found immigrant Chinese mothers in Europe or North America seldom breastfeed their babies [17, 18]. Thus, it seems important to identify factors influencing patterns of breastfeeding in immigrant populations, especially among Chinese immigrants.

In Ireland, the Chinese population is estimated at 60,000, and is potentially the largest (if not the second largest) minority ethnic community in Ireland [19, 20]. From 2011 to 2016, Chinese immigrants in Ireland increased by 9.1%, which represented the largest increase among all Asian groups [21]. A study using the Growing up in Ireland cohort of 11,092 mother-infant dyads found that although 91.5% Chinese immigrant mothers initiated breastfeeding, only 31.5% of infants were still breastfed at 9 months old [22]. Similarly, the Ireland Chinese Mother Survey involving 322 Chinese immigrant mothers found that although Chinese immigrants had a high breastfeeding initiation rate (75.6%) and any breastfeeding rate (87.2%), only 5.8% exclusively breastfed for 6 months [23, 24]. Given the rapidly growing Chinese immigrant population in Ireland and the low exclusive breastfeeding rates, there is a need to develop effective interventions and formulate a comprehensive strategy to promote exclusive breastfeeding among Chinese immigrants in Ireland.

A paper from our research group has been published previously to identify factors contributing to the success of exclusive breastfeeding among Chinese immigrant mothers living in Ireland who exclusively breastfed for 4 to 6 months and to describe barriers they had encountered [25]. However, this study did not include specific suggestions from Chinese immigrant mothers on how to improve and better promote breastfeeding among this population. The objective of the present study was to describe the views, opinions, and suggestions from Chinese immigrant mothers in Ireland to better inform interventions or policies to promote breastfeeding in the future among immigrant population.

## Methods

### Study design

Based on the phenomenological framework which exemplifies a constructionist epistemology [26], a qualitative approach was used to explore participants’ suggestions on exclusive breastfeeding promotion among Chinese immigrant mothers living in Ireland. Interviews were conducted between December 2009 and February 2010.

### Participants

Participants were recruited from the Ireland Chinese Mothers Survey (ICMS) Study. The ICMS included 322 Chinese women who were born in China and had been living in Ireland for more than 6 months. The original inclusion criteria can be found elsewhere [23, 24]. Participants from the Ireland Chinese Mothers Survey were invited to take part in the study if they met the following three criteria: 1) had given birth in Ireland; 2) breastfed successfully for over 6 months; and 3) exclusively breastfeed for 4 to 6 months in Ireland. All mothers (*n* = 16) who met the inclusion criteria were contacted via telephone calls. Two refused to participate due to time constraints.

### Setting and data collection

Before each interview, researchers informed the participants about the purpose and confidentiality of the study and obtained written consent. Researchers then conducted face-to-face, semi-structured interviews with the participants using an interview guide. The participants were prompted to share their views, opinions, and suggestions on 1) on how to breastfeed exclusively in Ireland; and 2) how the Irish government can improve exclusive breastfeeding rates in Ireland. Further probing questions were asked based on the interview context. The interview guide was inspired by a Hong Kong study which explored the relationship between situational variables and primipara mothers’ infant feeding behaviors [27, 28] and tailored to fit our study’s aims. The guide was then pilot tested with three Chinese mothers who had given birth in Ireland and was well understood by the participants.

Interviews were conducted in Mandarin and Cantonese (depending on the mother’s preference), recorded on a digital recorder and lasted for 45 to 125 min each time. Participants had the opportunity to ask questions and to withdraw from the study anytime during the interview. At the conclusion of each interview the participant’s demographic information were collected via a brief questionnaire and field notes were added. All recordings were transcribed verbatim into Mandarin by QZ. HC reviewed the taped interviews and validated the accuracy of the transcription of each interview.

### Data analysis

Thematic content analysis was used to analyze the data since it provides a flexible research tool that potentially provides rich, detailed and complex data [29].

Common themes were identified by HC and QZ following the guidelines proposed by Morse & Field [30]. Two researchers read the transcripts (not including the pilot test data) and coded the transcripts independently and systematically. Initial codes were generated and organized into categories. A tree diagram was developed to help organize the categories into a hierarchical structure [30] and the categories integrated into themes. HC and QZ then discussed the categories and themes to reach a consensus on the assignment of all themes and quotations that illustrated typical views. During the data analyses, field notes were reviewed against the transcripts. Quotations reflecting the participants’ opinions on exclusive breastfeeding promotion were also identified. Similar codes and themes generated from different interviews were gathered and opposite views identified. Preliminary results were provided to five participants for their verification. No problems were reported by these five participants.

## Results

### Sample characteristics

A total of 14 mothers between 24 and 54 years old (mean age: 34 years) were interviewed. At time of the study, the women had been living in Ireland for between three to 18 years (mean duration: 9 years). Eight mothers were primiparous, while six had two children, of which one mother had twins. Ten had achieved third-level education while four secondary or training school education. Over half of the participants were housewives or had part-time, non-professional jobs, while six were self-employed or had professional jobs. The majority had an annual family income over 30,000 Euro (before tax) and three had an annual family income of 15,000–30,000 Euro. All held positive attitudes towards exclusive breastfeeding because they believed that exclusive breastfeeding for 4 to 6 months benefited babies, mothers and society.

The results of the thematic analyses are summarized below. The four themes identified were: 1) being strong and getting support-suggestions for new immigrant mothers; 2) creating a supportive workplace-suggestions for employers; 3) addressing cultural differences and promoting breastfeeding in the hospital-suggestions for healthcare professionals; and 4) promoting societal and policy changes for breastfeeding-suggestions for the government.

### Being strong and getting support-suggestion for new immigrant mothers

Breastfeeding is not easy, even among immigrant mothers who managed to exclusively breastfeed their babies. All participants mentioned that strong willpower and persistence were key characteristics of their success with breastfeeding. One mom said, “*I was very determined when I decide to do this (breastfeeding), so I think strong determination is very important*.” (P1*).* Another mom said, *“it was inconvenient, but when I thought this (breastfeeding) was good for my baby, I would conquer any difficulties.”* (P4) Similarly, another said, “*first and foremost, you need to be strong, you must stay strong, you can complain, but do not give up. If I think this is the right thing to do, I will do it”* (P5)*.* Generally, it was suggested by the participants that new mothers should believe in themselves and remain positive during breastfeeding.

Many participants identified support received from family members, was crucial for new moms to initiate and continue breastfeeding. When probed about who provided the most support, many mentioned their parents. One mom said, “*I don’t think I can do this (exclusive breastfeeding) without the help of my parents. For example, when my babies cried in the middle of the night, I couldn’t pick them up because I was in the middle of pumping milk. At that time, my mom and dad would come and help taking care of them. If I was all by oneself, I don’t know how I could have done this*.” (P3) Besides parents, some moms emphasized the important role of their husband’s support. One indicated, “*my husband, he is very supportive for me to breastfeed my baby. For example, when I felt embarrassed to breastfed in public, he encouraged me and said it didn’t matter what other people say*.” (P1) Others mentioned that their husbands were able to adjust their working hours to fit their breastfeeding schedule so their wives didn’t have to feed alone.

In addition to family support, the participants also suggested new moms seek help from friends who had breastfeeding experience. One mother suggested, “*as they were experienced, they could understand your difficulties, tolerate the noise from the baby and provide timely help when necessary*.” (P14) When family members were not around to provide support, a good way for inexperienced new moms was to reach out to their friends who could offer practical help and emotional support.

### Creating a supportive workplace-suggestions for employers

Some working mothers suggested employers should find ways to support breastfeeding moms who came back to work following maternity leave. One mom suggested setting up nurseries within or near the workplace, “*the nurseries near my workplace were convenient for me to breastfeed, so this might be a way to support working moms who wish to continue breastfeeding*.” (P13) Besides setting up nurseries, other moms also suggested extending paid maternity leave, creating a private space in the workplace for breastfeeding moms who need to pump, and facilities to store breast milk. Notably, positive attitudes and mutual understanding from employers and colleagues were also mentioned as important factors in promoting breastfeeding in workplace. One mom said, “*I managed to continue breastfeeding exclusively after returning to work because my colleagues understood my situation well.*” (P3) This statement highlighted the importance of create a breastfeeding-friendly environment at the workplace.

### Addressing cultural differences and promoting breastfeeding in the hospital-suggestions for healthcare professionals

Generally, participants felt healthcare professionals in Irish hospitals and health centers should provide more information about breastfeeding. One mom observed, “*in Ireland, people pay less attention to breastfeeding (than in China).*” (P14) Similarly, another said, “*breastfeeding was seldom mentioned in medical advice. Doctors should encourage mothers to breastfeed.*” (P1) Some participants suggested that healthcare professionals should combine breastfeeding knowledge with breastfeeding practice during prenatal education classes for future breastfeeding initiation. “*Primipara were taught breastfeeding knowledge, but it’s difficult for them to operate in practice*. *.. Practice is needed to assist the understanding of theoretical knowledge.*” (P14).

When providing information, most participants suggested that healthcare professionals should address the language barriers encountered by Chinese immigrant mothers. One mom mentioned, “*in fact, there was still a language barrier. When I communicated with a healthcare professional, there were many professional terminologies which troubled me. So, I think maybe more Chinese nurses and midwives would be better.*” (P6). Other participants also indicated language-specific healthcare professionals should be recruited and trained to provide support and prenatal education for immigrant mothers as many first-generation Chinese immigrant mothers in Ireland had difficulties understanding English, especially professional terminologies. In addition, breastfeeding support groups targeting Chinese immigrant mothers to share experiences was also suggested.

Another suggestion for healthcare professionals was to address the conflicting information received by Chinese immigrant mothers from their country of origin and from Ireland. For example, one mother indicated that the treatment of breast milk jaundice by Chinese doctors and Irish doctors were completely different. “*My baby had jaundice due to breastfeeding. Chinese doctors advised to stop breastfeeding for one or two weeks, but Irish medical staff advised to increase breastfeeding frequency to promote infant digest[ion] and wet napp[ies].*” (P6) The participants suggested healthcare professional should be aware and consider such cultural differences and attempt to resolve the confusion on whether to continue breastfeeding.

### Promoting societal and policy changes for breastfeeding-suggestions for the government

Most participants suggested that government agencies increase and enrich breastfeeding publicity by various means. For example, enhanced publicity at clinics, within the community and on television as possible avenues for mothers to better understand the benefits of breastfeeding. One mom mentioned, “*the government still need to increase the publicity to let more mothers know that breast milk is better than bottles.*” (P9) In order to reach the target audience, many participants suggested that breastfeeding information should be easily available Chinese languages and various forms such as brochures and books. “*Add some Chinese information on breastfeeding.*” (P12). “*Publicity can be made through newspapers and television.*” (P5). “*Publicizing breastfeeding to Chinese immigrants by brochures may be better, because they don’t like to join clubs.*” (P6) Regarding the content of breastfeeding promotional materials, the participants identified some topics such as emphasizing that breastfeeding is natural, necessary and worthwhile when compared to infant formula feeding. “*I think you can compare breast milk with formula, and present in a table.*” (P9) Such strategies may help moms to understand the pros and cons of breastfeeding and infant formula feeding. Other topics included providing education on benefits of breastfeeding, correcting breastfeeding misconceptions and discussing solutions to breastfeeding problems such as for mothers taking medication.

Besides increasing publicity, some participants suggested that Irish governmental agencies should amend the laws and regulations to provide more support for breastfeeding Chinese mothers. One mom suggested the provision of financial subsidies during maternity leave. “*The government could give appropriate subsidies for breastfeeding, such as dozens of Euros a month.*” (P11) Others suggested developing new social security policies for Chinese immigrants such as narrowing the gap between social welfare benefit entitlements between Irish and immigrant residents. “*As there is a big gap of social welfare between immigrants and Irish, I do hope that Ireland could provide special social welfare policy for immigrants.*” (P14) Providing a longer-term Irish visa for Chinese mothers in Ireland for education-related reasons was also suggested to ensure they breastfed for the first 6 months after birth. A newly arrived mother said, “*I attended a language class in the second month after delivery [because I was on a] student visa which caused difficulties in exclusive breastfeeding.*” (P14).

Another common suggestion was that government agencies should develop policies to urge public places, such as shopping malls, to set up better equipped breastfeeding facilities. One mom said, “*There were many places for nappy changing, but breastfeeding facilities are not enough*.” (P5). Others suggested equipping these rooms with seats, water and breast milk heaters to enhance breastfeeding experiences.

## Discussion

This is the first study exploring Chinese immigrant mothers’ suggestions on the promotion of exclusive breastfeeding in Ireland. This paper complements an earlier paper from our group which identified facilitators and barriers to breastfeeding among this ethnic group [25]. The findings of the current study provide breastfeeding promotion suggestions for Chinese immigrant mothers, employers, healthcare professionals and Irish governmental agencies. These suggestions might inspire other countries with low breastfeeding rates, as few studies had explored immigrants’ opinions on improving exclusive breastfeeding rates in host countries.

### Suggestions for new immigrant mothers

The important role of maternal attitudes towards breastfeeding has been reported in a number of studies [31–34]. Consistently, our findings implied that Chinese mothers’ strong willpower to exclusively breastfeed was important to the success of optimal feeding practices. Maternal attitudes might especially be true for the first generation of Chinese immigrant mothers because of traditional beliefs that mothers are strong, persistent and should do whatever is best for their off-spring’s well-being. For new mothers, reinforcing traditional positive attitudes and their ability to breastfeed along with increasing self-efficacy, appears to be crucial to increasing breastfeeding rates among new mothers.

In addition to maternal attitudes, previous studies have identified family support as an important factor in supporting exclusively breastfeeding [35–37]. The findings of this study also suggested that new mothers turn to their family members for support during lactation. Notably, besides parents, the encouragement and cooperation of husbands during lactation were crucial to the success of exclusively breastfeeding. It was reported that most Chinese-Irish families were nuclear families (a family group consisting of two parents and their children) where the husband was the main source of financial support [23, 37]. The husband’s busy working schedule may have contributed to a lack of involvement or support of exclusively breastfeeding. Indeed, a previous study confirmed that increasing fathers’ involvement during an infants’ first year of life may improve breastfeeding duration up to 6 months of age [38]. Other studies have also reported that husbands’ positive attitudes and support are associated with longer breastfeeding duration [39, 40]. To address these issues and promote breastfeeding in Ireland, relevant policies have been developed in Ireland. As of November 2019, under the new employment legislation, both new mothers and fathers are entitled to 2 weeks extra parental leave on top of current maternity/paternity leave entitlements [41]. This policy may help fathers to be more involved and to better support breastfeeding.

### Suggestions for employers

Chinese immigrant mothers in this study considered a supportive working environment a key factor for the success of exclusive breastfeeding, especially the need to establish relevant policies on extending maternity leave. Previous studies in other countries have shown that immigrant women are more likely to return to work earlier for financial reasons, despite their intention to breastfeed for 6 months or longer [42, 43]. Therefore, as suggested by our study participants, ensuring a sufficient period of paid maternity leave may help improve the duration of breastfeeding among immigrant mothers. To date, some relevant policies, such as the Maternity Protection (Amendment) Act (2004), have been released to ensure the rights of breastfeeding mothers in Ireland [44]. These policies allow breastfeeding mothers maternity leave of no less than 18 consecutive working weeks and to breastfeed during working hours without loss of pay [44, 45]. However, our study participants rarely mentioned these policies, possibly due to their unawareness of this entitlement. It should be noted that these policies were updated as the Parent’s Leave and Benefit Act 2019 [41], which allowed for a total of 26 weeks paid maternity leave. Employers need to provide and explain these updated policies in detail to ensure that immigrant mothers are aware of their breastfeeding rights in the work place.

Further, previous studies have shown an unfavorable workplace environment may be significantly associated with the cessation of breastfeeding [28, 46]. The findings from our study also suggest the importance of a breastfeeding-friendly workplace (e.g., nurseries, breastfeeding rooms) and further suggest that employers set up a private space and a safe and clean facility for breast milk storage. It should also be noted that under Irish law, employers are not obliged to provide breastfeeding facilities in the workplace if the provision of such facilities involves considerable costs [44].Consequently, there is a need to explore ways to reduce the cost of providing workplace breastfeeding facilities/spaces and/or incentives for employers to adopt such policies.

### Suggestions for healthcare professionals

Breastfeeding counseling provided by healthcare professionals is also a useful source for obtaining breastfeeding knowledge [43, 47]. Previous research has established the importance of healthcare professionals’ attitudes and advice on exclusive breastfeeding rates among Chinese immigrants [12, 43]. Participants in our study felt that healthcare professionals did not sufficiently stress the importance of breastfeeding in the hospital setting, and needed to talk more explicitly about the practice with immigrant mothers.

It should be noted that the interviews for this study were collected 10 years ago and changes have been made to the Irish healthcare system over the past decade. According to 2015 Breastfeeding Policy for Primary Care Teams and Community Health Care Settings developed by Irish Health Service Executive (HSE) [48], all staff involved in the care of pregnant women, infants and young children should support and enable mothers to breastfeed exclusively for 6 months. This policy indicates training should be provided for all health workers on the knowledge and skills necessary to implement the breastfeeding policy; and all health workers should discuss with pregnant women and their families the importance and management of breastfeeding [48]. In 2020, Sullivan et al. published a paper examining breastfeeding beliefs among Polish-born mothers in Ireland which suggested that health professionals were very “pushing” about breastfeeding [49]. This outcome may be the result of the efforts made to improve breastfeeding training train among healthcare professionals. Additionally, the National Standards for Antenatal Education in Ireland was published in 2020 [50]. This document provides information on the content, mode of delivery and training resources for the development of a high-quality antenatal education program. It also set up the standards for such programs in Ireland [50]. However, no specific section or reference to immigrant mothers was found.

Despite the promising results from training healthcare professionals in Ireland, limited improvements have been shown to address the language and cultural differences encountered by immigrant mothers. Our study suggested that the language barrier was a great impedance to obtain breastfeeding relevant information by Chinese immigrant mothers. This finding is consistent with many other studies among immigrant populations [18, 23, 43]. A translation service might be used to overcome this barrier. However, according to another study among immigrant women in Ireland, the lack of access to trained interpreters continued to be a problem and was often limited in the hospitals due to cost [51]. Therefore, the recruitment of multilingual staff is needed in the healthcare system. Besides language problems, multilingual healthcare professionals may come from the same or similar culture and have a better understanding of the cultural differences encountered by immigrant mothers. Consequently, they might be able to clarify specific misconceptions or conflicted information received by immigrant mothers. Currently, no data have been published on whether the recruitment of multilingual staff has been increased since the time of this study. Future research should address this gap and collect information on how the Irish healthcare system deals with language problems in the hospitals.

### Suggestions for government agencies

Nearly half of our participants reflected that government agencies should initiate more societal changes to promote breastfeeding among immigrants in Ireland. Such moves should include increasing publicity on breastfeeding and making breastfeeding messages more visible via brochures, books or television. To the author’s best knowledge, information in Chinese languages on the Irish government website remains limited with, so far, only one flyer called “Feeding cues” is available in Chinese on the HSE website [52]. More language-specific material on topics proposed by our study participants such as comparisons between breastfeeding and infant formula feeding, the benefits of breastfeeding, breastfeeding misconceptions and solutions to breastfeeding problems, should be developed and made available to immigrant mothers.

Although not mentioned by the participants, a hotline might be another effective way to increase breastfeeding publicity among immigrant populations, as indicated by one study conducted in Chinese-Canadian population [53]. In Ireland, no hotline has been established, but an email service called “ask the expert on-line lactation consultant service” is available on HSE websites and has been providing support for breastfeeding mothers who have questions or need to join a local breastfeeding support group [54]. However, it is unclear whether this email service has multilingual agents to respond to the immigrant population from non-English speaking countries. Further exploration on the effectiveness of this email service in improving the breastfeeding rates in Ireland immigrant population is needed.

Other suggestions proposed by our study participants included setting up better equipped, specific breastfeeding rooms in public places, especially shopping malls. Although the Equal Status Act (2000) prevents discrimination and harassment of breastfeeding in public [45], mothers may still feel embarrassed and the need some privacy when it comes to breastfeeding. Certain policies should be put in place to help create a safe place in public for breastfeeding moms. Additionally, the participants suggested several other policy changes, including developing new social security policies for Chinese immigrants, narrowing the gap of social welfare benefits between Irish and immigrant residents, and providing a longer-term Irish visa for breastfeeding moms. These issues have not been previously documented and currently there are no such policies in place. Such policies could be of future consideration for the Irish Immigrant Council of Ireland.

Several limitations should be kept in mind when interpreting the results of this study. First, the interviews were collected 10 years ago, and suggestions from the findings may not be relevant today. However, to the best of the author’s knowledge following a thorough search online, little has been done to address suggestions such as recruiting multilingual healthcare professionals or establishing policies to support breastfeeding among immigrant population. Further research should also be conducted to explore the efficacy of any legislative changes in support of breastfeeding and their impact on the experiences of immigrant mothers. Additionally, the generalizability of the findings is limited given this study involved only a small sample of Chinese immigrant mothers. Consequently, it is possible that our results may not be reflect the experiences of the general population of Chinese immigrant mothers. Additionally, our results may not be fully comprehensive, as we only included mothers who exclusively breastfed for at least 4 months. The rationale for this restriction was to document the opinions of mothers whose exclusive breastfeeding experience was successful and positive. Nonetheless, constructive opinions from immigrants’ families and husbands and mothers who breastfed for less than 4 months are warranted in further studies.

## Conclusions

This study explored the opinions and suggestions on the promotion of exclusive breastfeeding in Ireland among fourteen Chinese immigrant mothers who had a successful experience. Specific suggestions for new mothers, employers, healthcare professionals and the Irish government were identified. Findings from this study may help researchers understand needs of Chinese immigrant mothers to successfully exclusively breastfeed and may also have implications for breastfeeding interventions and policy changes that promote breastfeeding among immigrant populations in Ireland and/or other countries.

## Data Availability

The data supporting the conclusions of this article are available from the corresponding author on reasonable request.

## Abbreviations

HSE: Health Service Executive
ICMS: the Ireland Chinese Mothers Survey Study
WHO: World Health Organization
